# Loss of *Calretinin* in L5a impairs the formation of the barrel cortex leading to abnormal whisker-mediated behaviors

**DOI:** 10.1186/s13041-021-00775-w

**Published:** 2021-04-12

**Authors:** Mingzhao Su, Junhua Liu, Baocong Yu, Kaixing Zhou, Congli Sun, Mengjie Yang, Chunjie Zhao

**Affiliations:** Key Laboratory of Developmental Genes and Human Diseases, Ministry of Education, School of Medicine, Southeast University, Nanjing, 210009 China

**Keywords:** Calretinin, Layer 5a pyramidal neurons, Barrel cortex, Lemniscal pathway, Paralemniscal pathway, Exploratory behavior, Tactile sensation behavior

## Abstract

The rodent whisker-barrel cortex system has been established as an ideal model for studying sensory information integration. The barrel cortex consists of barrel and septa columns that receive information input from the lemniscal and paralemniscal pathways, respectively. Layer 5a is involved in both barrel and septa circuits and play a key role in information integration. However, the role of layer 5a in the development of the barrel cortex remains unclear. Previously, we found that calretinin is dynamically expressed in layer 5a. In this study, we analyzed *calretinin* KO mice and found that the dendritic complexity and length of layer 5a pyramidal neurons were significantly decreased after *calretinin* ablation. The membrane excitability and excitatory synaptic transmission of layer 5a neurons were increased. Consequently, the organization of the barrels was impaired. Moreover, layer 4 spiny stellate cells were not able to properly gather, leading to abnormal formation of barrel walls as the ratio of barrel/septum size obviously decreased. *Calretinin* KO mice exhibited deficits in exploratory and whisker-associated tactile behaviors as well as social novelty preference. Our study expands our knowledge of layer 5a pyramidal neurons in the formation of barrel walls and deepens the understanding of the development of the whisker-barrel cortex system.

**Supplementary Information:**

The online version contains supplementary material available at 10.1186/s13041-021-00775-w.

## Introduction

Rodents use their whiskers to explore the presence and location of objects when moving through a nocturnal environment and evolved a whisker-barrel cortex system [1–5]. As the information-receiving region, the barrel cortex plays a crucial role in integrating information resources and coordinating the movement of the whiskers and determines the function of the entire system [2, 6–9]. The barrel cortex consists of barrel and septa columns that receive various input signals through distinct pathways. The lemniscal pathway transmits whisker-specific signals to homologous barrel columns, and the paralemniscal pathway transmits multiwhisker signals to both barrel and septa columns [9–13]. The integration of information from both the lemniscal and paralemniscal pathways in the barrel cortex is a prerequisite for precise object recognition [6, 14].

As the main target of the paralemniscal pathway, layer 5a (L5a) is involved in both barrel and septa circuits and is considered to be an important component of information integration for the lemniscal and paralemniscal pathways [10, 15–17]. L5a pyramidal neurons directly accept the information inputs from the paralemniscal pathway and indirectly accept the information inputs from the lemniscal pathway. By neuronal tracer techniques, it has been observed that dendrites of L5a pyramidal neurons have a larger span and can establish synaptic connections with layer 2/3 (L2/3) and layer 4 (L4) neurons [18–20]. The earliest input to L5a pyramidal neurons is provided by L4 spiny stellate cells, which is then followed by an asynchronous L2/3 input [20]. The information from L4 spiny stellate cells is strong direct, monosynaptic [21]. Meanwhile, as the main information input layers of the paralemniscal pathway, layer 1 (L1) and L5a are closely related to each other [6, 18, 22]. However, the effects of L5a pyramidal neurons on the development of the barrel cortex and related behaviors need to be further elucidated.

Calcium ions (Ca^2+^) participate in a series of physiological functions, including gene transcription, enzyme activity, ionic channel permeability, and neurotransmitter release [23–25]. The precise control of Ca^2+^ concentration and spatial location is a prerequisite for accomplishing these tasks. This control is a function of the balance of Ca^2+^ transport mechanisms across the plasma membrane, the storage and mobilization of Ca^2+^ from intracellular stores, and the actions of a multitude of calcium-binding proteins (CaBPs) located throughout the cytoplasm [25]. CALRETININ (CR) [26], which belongs to the EF-hand Ca^2+^-binding protein family [27, 28], is a well-known Ca^2+^ buffer influencing spatiotemporal Ca^2+^ transients within the cytosol [29]. Previous studies have reported that CR, as a Ca^2+^ sensor [28], is also required for signaling cascades in response to intracellular Ca^2+^ transients.

Previously, we showed that CR is dynamically expressed in L5a pyramidal neurons in the developing barrel cortex and displays a unique predominantly serrated pattern that mirrors the presynaptic projection pattern of the posterior medial nucleus (Pom) to L5a [10, 30]. In this study, by analyzing *calretinin* knockout (*Cr* KO) mice, we found that loss of *Cr* results in a reduced complexity of L5a pyramidal neuron dendrites, which leads to abnormal formation of the barrel wall and subsequently impairs the barrel and septa microcircuits. *Cr* KO mice exhibit abnormal exploratory and tactile sensation behaviors. Our results provide evidence that L5a pyramidal neurons direct the formation of the barrel wall during the development of the barrel cortex.

## Materials and methods

### Animals

The *CR-CreER* line, which was designed to both abolish the *Cr* gene and express an inducible site-specific Cre recombinase (stock number: 013730), was purchased from the Jackson Laboratory. The *AI9-RFP* (stock number: 007905) line was introduced to trace CR^+^ L5a neurons [31]. *Cr* KO mice were obtained by intercrossing *CR*^*CreER/*+^ mice. WT and heterozygous mice were used as controls. The day of birth was defined as postnatal day P0. Tamoxifen induction was performed at P5 and P8. Mice were bred in the animal facility at Southeast University. All of the experiments were performed according to the guidelines approved by Southeast University.

### Immunostaining and morphological analysis

Immunostaining was then performed as previously described [32, 33]. Mice were transcardially perfused with 4% paraformaldehyde (PFA), postfixed at 4 °C overnight, cryoprotected in 30% sucrose, and embedded in optimum cutting temperature (OCT) compound. Sections (25 μm thick) were obtained by a Leica cryostat (CM 3050S). Immunostaining was performed as previously described. Rabbit anti-calretinin (Millipore, AB5054, 1:1000), mouse anti-RFP (Abcam, ab125244, 1:500) and mouse anti-vGlut2 (Synaptic System, 135311, 1:500) were used as primary antibodies, and Alexa Fluor 488 goat anti-rabbit IgG (Molecular Probes, A11008, 1:500) and Alexa Fluor 633 goat anti-mouse IgG (Molecular Probes, A21050, 1:500) were used as secondary antibodies. Before coverslips were applied, the slides were incubated with DAPI (Sigma, D9564, 1:1000) for 15 min. Pictures were captured by a confocal microscope (Olympus, FV1000).

Images of dendritic arbors were captured under a 40 × objective lens with a confocal microscope (Olympus, FV1000) in Z-stack mode (Additional file 1) [34]. Neuron morphology was traced manually using the NeuronJ plugin in ImageJ software. Standard morphometric analysis (Sholl analysis) was conducted as described earlier. Significance was determined by a two-way repeated-measures analysis of variance (RM 2-ANOVA; genotype and circle radius as factors).

### Western blot

Somatosensory cortex homogenates were collected at P8 and prepared as described previously [34]. Protein samples were run on SDS-PAGE and transferred to cellulose acetate membranes. After incubation in TBS containing 5‰ Tween 20 and 5% milk for 1 h at room temperature (RT), the membranes were incubated with primary antibody (rabbit anti-Calretinin, Millipore, AB5054, 1:2000) at 4 °C overnight. After washing in 5‰ Tween 20 in TBS for 30 min, the membranes were incubated with secondary antibody (HRP-linked anti-rabbit IgG, Cell Signaling Technology, 7074S, 1:5000) in TBS buffer for 1 h at room temperature, and immunoreactive bands were visualized with an ECL kit (Thermo Scientific). Quantitative analysis was performed with ImageJ software. The intensity of bands was normalized to the intensity of the corresponding β-tubulin band. An unpaired Student’s t-test was used to determine the significance.

### Nissl staining

The brain slices were removed from the ultralow temperature refrigerator and allowed to dry at room temperature. Then, the slices were immersed in distilled water. After 2 min, the slices were removed and placed in Nissl staining solution for 10 min. After washing with distilled water for 10 min, the slices were immersed in 95% alcohol for 5 s. The slices were dipped in xylene for 5 min and sealed with rhamsan gum (Additional file 1) [35].

### Electrophysiology

#### Slice preparation

P18 to P20 brain slices were used for electrophysiological experiments. Briefly, mice were anesthetized by inhalation of isoflurane, and the brains were quickly removed and immersed in precooled artificial cerebrospinal fluid (ACSF) containing (in mM) 125 NaCl, 2.5 KCl, 1.25 NaH_2_PO_4_, 26 NaHCO_3_, 1 CaCl_2_, 6 MgCl_2_, and 10 glucose. Coronal slices at a thickness of 350 μm were obtained using a vibrating microtome (Leica Microsystems, VT1200s) [36]. The slices were incubated in a chamber at 35 °C for 30 min and then maintained at room temperature (22 °C) for at least 1 h before recording.

### Electrophysiological recording

Electrophysiology was performed as described previously [35, 37]. Briefly, brain slices were placed in the recording chamber and completely submerged in ACSF (bubbled with 95% O_2_/5% CO_2_). Whole-cell recordings were performed on RFP^+^ neurons in L5a. The RFP^+^ neurons were detected by a fluorescence microscope. The recording was aided with infrared optics using an upright microscope equipped with a × 40 water-immersion lens (Olympus BX51W1, Japan) and an infrared-sensitive CCD camera. The pipette (input resistance: 3–6 MΩ) solution contained the following (in mM): 125 potassium D-gluconate, 8 NaCl, 0.2 EGTA, 10 HEPES, 2 Mg-ATP, 0.3 Na-GTP. Patch pipettes were pulled on a horizontal pipette puller (P-97, Sutter Instrument). Series resistances were usually 15–30 MΩ upon break-in and were compensated by 70%. RFP^+^ neurons with stable series resistance (20% change throughout the recording) were used for analysis. Data were recorded by an Axon patch 700B amplifier (Molecular Devices), low-pass filtered at 2 kHz and digitally sampled at 10 kHz online and analyzed offline with Clampfit software (Molecular Devices). To characterize the intrinsic membrane properties of neurons, spiking patterns were recorded in the current-clamp configuration by injecting a series of current pulses (400 ms duration, − 50 to 300 pA intensity with an increment of 50 pA). The following parameters were measured to characterize neuronal membrane properties: the resting membrane potential was recorded immediately after the rupture of the neuronal membrane; the action potential current threshold was defined as the first 400 ms rectangular current injection that elicited a spike; the input resistance was determined by measuring the voltage change in response to a hyperpolarizing current pulse; the amplitude of afterhyperpolarization (AHP) was measured as the distance between the threshold and the most negative membrane potential following the spike of the first action potential evoked by the first current step evoking action potentials; and the spike width was measured at half the height between the threshold and peak action potentials. To isolate miniature EPSCs (mEPSCs), tetrodotoxin (TTX, MCE, 1 μM, to block sodium current) and bicuculline (BMI, Sigma-Aldrich, 14,343, 10 μM, to block GABA receptors) were added to the bath solution. mEPSCs were analyzed using the Mini Analysis Program (Version 6.0.3, Synaptosoft), and all events were detected above a threshold of 5 pA.

### Behavioral tests

All behavioral tests were performed using groups of 2- to 3-month-old littermate male mice on a C57BL/6 J background [38]. 3–5 mice were housed in a same cage regardless of its genotype. All behavioral assays were performed blind to genotypes. The tests were performed in the following order: open-field tests, elevated O-maze tests, elevated plus maze tests, novel object investigation tests, S curve tests, sticky paper tests, texture discrimination tests, gap crossing tests, social behavior tests. The mice were handled for 5 days before the beginning of behavioral testing and left to acclimate in the testing rooms for at least 30 min before the experiments. All tests had an interval of at least 1 day between each other. Open-field tests, novel object investigation tests, elevated O-maze tests, elevated plus maze tests, S curve tests, texture discrimination tests and social behavior tests were conducted under red lighting; sticky paper tests and gap crossing tests were conducted under infrared lighting [39]. All videos were taken using high-resolution digital cameras and analyzed by EthoVision software (Noldus) in a double-blinded manner.

### Open-field test

Mice were introduced into the center of the chamber (40 × 40 × 40 cm) at the beginning of the test. Their movements were recorded with a video camera for 30 min.

### Novel object investigation test

Mice were placed into the open-field box for 10 min of locomotor activity. Then, a novel object was placed in the center of the open-field box. The mice were allowed to explore for 10 min, and their locomotor activity was recorded and analyzed [40].

### Elevated plus maze and elevated O-maze tests

Both the elevated plus maze and O-maze were 1 m in height. The elevated plus maze consisted of a central platform (6 × 6 cm) with two opposing open arms (30 × 6 × 0.5 cm) and two arms enclosed by Plexiglas walls (30 × 6 × 15 cm). The O-maze consisted of a 6 cm-wide ring. The outer diameter of the equipment was 45 cm, containing two equal open sections and two closed sections. The mice were placed at the boundary between the open and closed sections and tracked for 10 min with EthoVision software [37].

### S curve test

The size of the S curve was 6 × 120 × 15 cm; one end was closed, and the other end was open. Mice were introduced into the closed end. The time they spent reaching the destination was recorded as incubation, and the time before they left the curve was recorded as total time.

### Texture discrimination test

Similar to the novel object investigation test, two glass bottles (3 cm in diameter and 5 cm in height), one of which was wrapped in sandpaper, were placed in the opposite corners of the open-field box. Mice were then introduced into the center of the box and allowed to explore different textures with their whiskers for 10 min.

### Sticky paper test

We performed the sticky paper test in the home cage [41]. Two adhesive-backed papers (0.5 cm in diameter) were placed on the palmar surface of the hind paws, and the latency of the first reaction to the stimulus was recorded as incubation.

### Gap crossing test

The gap crossing test consisted of a series of trials during which mice were required to cross gaps of variable distance [42]. The mice were placed on an elevated lane (6 cm in diameter). The lane connected to a safe platform. The distance between the lane and the platform was changed from 0 to 7 cm in trials by 0.5 cm increments. The distances that the mice were able to cross were recorded.

### Social behavior test

The social behavior test consisted of three phases [34, 38]. In the first phase, the test mouse was placed in the middle of a three-chambered box (40 × 20 cm) with open middle sections on each of the transparent dividing walls. There were two small containers in the left or right chamber. The mouse was allowed to explore all three chambers for 5 min for habituation. After 5 min, a stranger male mouse was introduced into one of the two containers, the test mice were placed in the middle and allowed to explore the new environment freely for 10 min. In the last phase, a new stranger male mouse was placed in the last empty container, and the test mice were also placed in the middle and allowed to explore the environment freely for 10 min. We measured the exploration time in each side of the three-chamber box during the three phases. All apparatus chambers were cleaned with 75% alcohol and dried with a pledget between trials.

### Statistical analysis

All data are presented as the mean ± standard error of the mean (SEM) and were analyzed using GraphPad Prism 8.0 software. Student’s two-tailed t-tests were used for analysis of two experimental groups. One-way ANOVA with Tukey’s post hoc test was used when more than two groups were compared (Additional file 2). Statistical significance was defined at P < 0.05 and is presented as *P < 0.05, **P < 0.01, ***P < 0.001, ****P < 0.0001.

## Results

### Loss of *Cr* results in decreased dendritic complexity of L5a pyramidal neurons

**Fig. 1:**
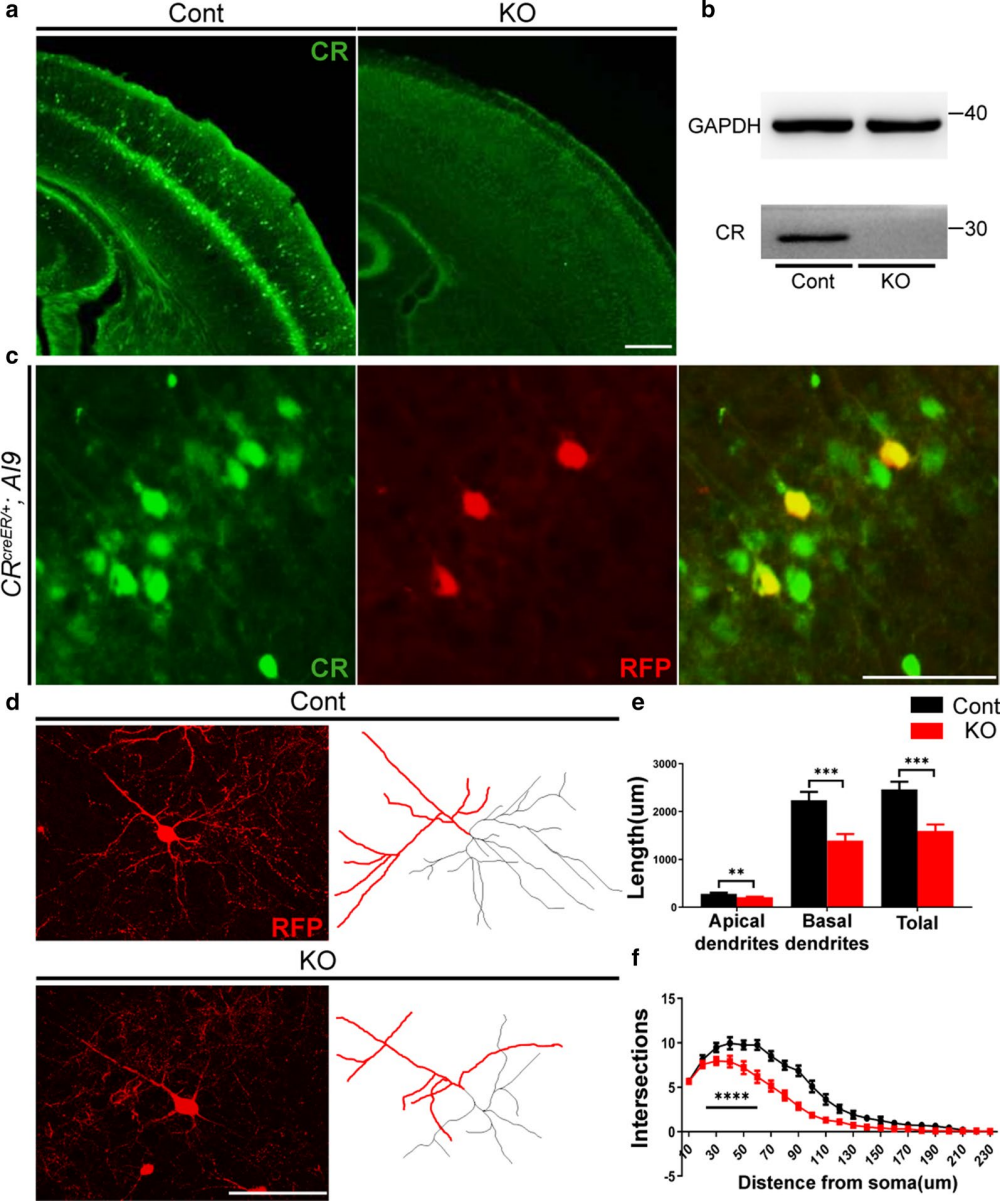
Loss of *Cr* impairs the dendritic architecture of L5a pyramidal neurons. **a** Immunostaining of CR in the P8 barrel cortex showed that *Cr* was efficiently disrupted in *Cr* KO mice. **b** Western blot analysis of cortex samples indicated that CR was completely deleted at P8 in *Cr* KO mice. **c** Immunostaining against RFP in the barrel cortex of *CR*^*CreER;Ai9*^ mice showed that CR^+^ L5a pyramidal neurons were labeled by RFP. **d** Immunostaining against RFP combined with morphologic tracing with ImageJ software (apical dendrites are indicated in red; basal dendrites are indicated in black) showed that in *Cr* KO mice, the complexity of dendrites of RFP^+^ neurons was significantly reduced in the P20 barrel cortex. **e** The lengths of apical dendrites, basal dendrites and total dendrites were significantly reduced in *Cr* KO neurons at P20 (Cont, 21 cells from 3 mice; KO, 27 cells from 4 mice). **f** Sholl analysis of L5a RFP^+^ neurons at P20 showing that, compared with that in control mice, the complexity of dendrites in *Cr* KO mice was markedly decreased (Cont, 21 cells from 3 mice; KO, 27 cells from 4 mice). Data are presented as the mean ± SEM; E, unpaired Student’s t-test. F, two-way ANOVA, distance x group, repeated measure. **p < 0.01; ***p < 0.001; ****p < 0.0001. Scale bars: **a** 500 μm; **c**, **d** 100 μm

P0–P15 is a key time window for the experience-dependent development of the barrel cortex [22, 43–46]. Interestingly, we previously observed dynamic expression of *Cr* in L5a pyramidal neurons during this period [30]. We then employed *Cr* KO mice to explore the role of CR in the development of L5a pyramidal neurons. We first confirmed the disruption of CR in the developing barrel cortex at P8 by immunohistochemical staining (Fig. 1a) and Western blotting (Fig. 1b). Since an inducible Cre recombinase (CreER) was targeted to the *Cr* locus, the *AI9-RFP* line was next introduced to trace CR^+^ L5a pyramidal neurons (Fig. 1c). We analyzed the dendritic morphology of RFP^+^ neurons at P20 and found that control mice exhibited a typical morphology of L5a pyramidal neurons with an apical dendrite extending to L1 and several basal dendrites stretching to the sides and deeper [20]; however, the complexity of basal dendrites was significantly reduced in *Cr* KO mice (Fig. 1d). We measured the dendritic length of RFP^+^ neurons and detected significant reductions in the apical, basal and total dendritic lengths in *Cr* KO mice compared with those in control mice (Fig. 1e). The apical dendritic length decreased by approximately 25%, and the basal and total dendritic lengths decreased by approximately 35%. We also observed a marked decrease in dendritic complexity in *Cr* KO mice (Fig. 1f). These results suggest that ablation of *Cr* led to abnormal dendritic development of L5a pyramidal neurons.

### Impaired organization of barrels and abnormal formation of the barrel wall in *Cr* KO mice

**Fig. 2:**
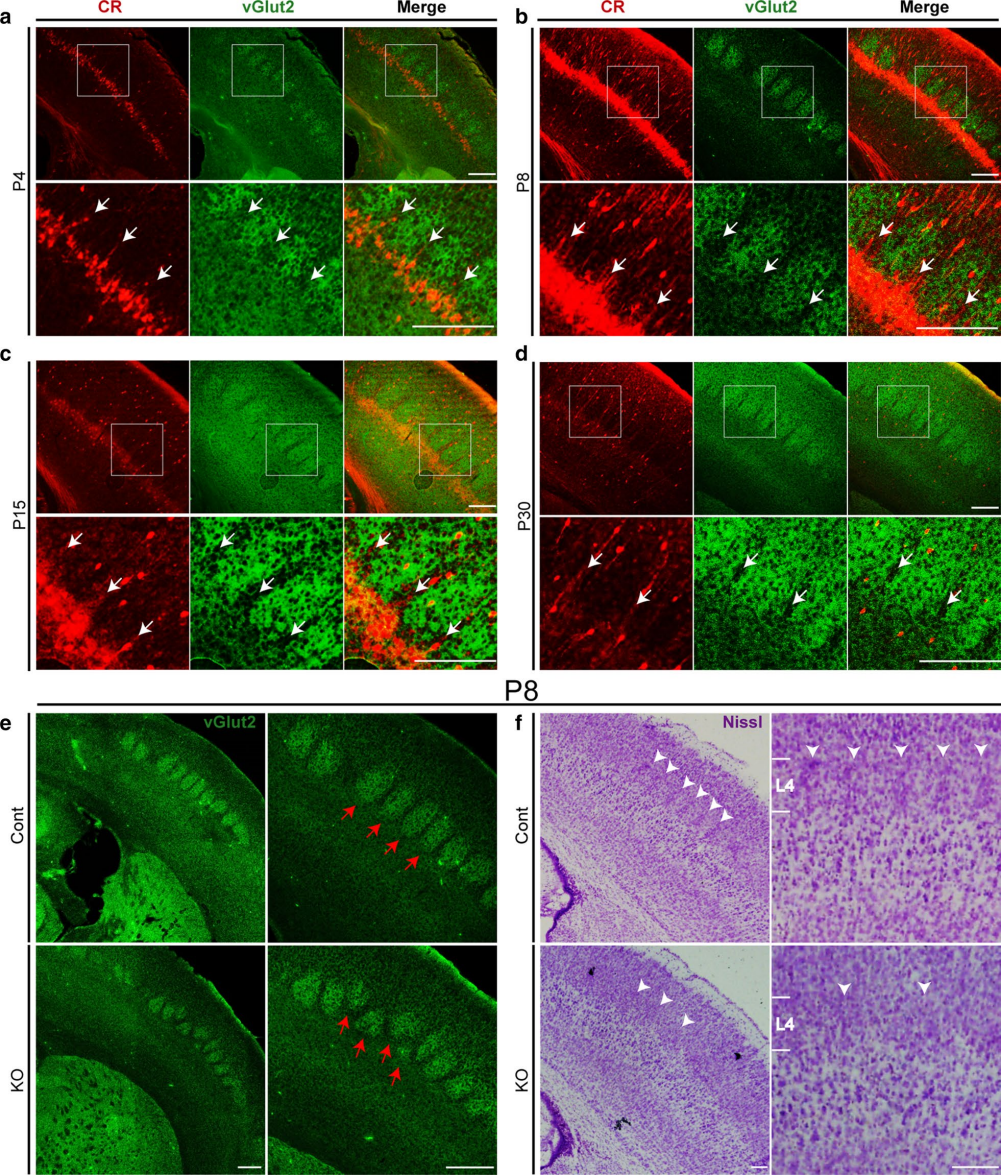
*Cr* ablation in L5a impairs the organization of barrels. **a**–**d** Immunostaining of CR and vGlut2 showing the distribution of CR^+^ L5a pyramidal neurons and barrels. During the development of the barrel cortex, CR^+^ L5a pyramidal neurons displayed a distinct serrated alignment pattern (high-magnification views of the boxed regions), and their dendrites extended towards the septa (white arrows: the dendrites formed septa-like structures). At P4 (**a**), the pattern was distinct. The peak expression level of CR was observed at P8 (**b**), and the pattern became more distinct and was maintained until P15 (**c**). The expression of CR in L5a pyramidal neurons was indistinct at P30 (**d**), and the pattern almost disappeared. **e** Immunostaining of vGlut2 in the P8 barrel cortex showed that the organization of the barrels was disordered in *Cr* KO mice (indicated by the red arrows; Cont, n = 4; KO, n = 4). **f** Nissl staining at P8 showing that, in contrast to the clear thin barrel wall observed in the control barrel cortex, abnormally gathered spiny stellate cells were observed in L4 of the *Cr* KO cortex (indicated by the white arrowheads; Cont, n = 4; KO, n = 4). Scale bars: **a**–**d** 200 μm; **e** 500 μm; **f** 100 μm

To investigate the contribution of L5a to the development of the barrel cortex, we carefully profiled the morphological changes of L5a pyramidal neurons during the time window of P0–P30 by double immunostaining of CR and vGlut2, a marker commonly used to label cortical barrels [47]. At P4, the CR^+^ L5a pyramidal neurons displayed a serrated pattern of alignment underneath the barrels, with their dendrites starting to extend towards the intervals between barrels (Fig. 2a). From P8 to P15, the serrated alignment pattern of the CR^+^ L5a cell bodies became more distinct, and more dendrites were observed in the intervals and formed septa-like structures (Fig. 2b, c) [48]. From P15–P30, as the maturation of the barrel cortex proceeded, the expression level of *Cr* in L5a gradually decreased. Until P30, the barrels in L4 were well developed (Fig. 2d). The expression of *Cr* was decreased to a very low level both in L5a cell bodies and in septa-like structures (Fig. 2d).

L4 is the main recipient layer of the whisker-barrel cortex system, spiny stellate neurons are the main excitatory neurons in L4 in the barrel cortex and are primarily accumulate at the barrel edge. L4 spiny stellate neurons expand their dendrites predominantly within a single corresponding barrel to form synapses with thalamocortical axons (TCAs) termini. This strong asymmetric dendritic orientation is a critical morphological basis of the precise one-to-one functional relationship between whiskers and barrels. L5a is the main output layer, and the two layers are monosynaptically connected [2, 6, 49]. The connections of L4 to L5a form a “short circuit” between afferent signals to the cortex and efferent signals that leave the barrel cortex from L5a [8]. To assess the effects of abnormal L5a dendrite on the development of barrel/septum microcircuitry, we examined the barrel cortex at P8. As shown in Fig. 2e, in contrast to the regularly organized barrels at L4 in control mice, we found barrels with disrupted organization in *Cr* KO mice, with some barrels deviating from their intrinsic level.

During the development of the barrel cortex, TCAs reach L4 around P1, and clusters of axons can be detected in the barrel cortex at P3, reflecting an increase in the complexity of axonal endings [22, 50]. Subsequently, L4 spiny stellate cells reorganize around TCA axonal clusters to form barrel walls [45]. At P7, barrel walls are clearly visible. L5a pyramidal neurons are reported to preferentially connect with barrel walls, but whether L5a is required for the formation of barrel walls remains unclear. In our control mice, consistent with previous reports, at P8, L4 spiny stellate cells gathered to form a clear barrel wall between barrels, as shown by Nissl staining. Interestingly, we found that barrel wall-like structures were completely missing in the entire *Cr* KO barrel cortex (Fig. 2f). These data indicate that in addition to the TCAs endings, the L5a CR^+^ dendritic tree may also contribute to the organization of barrels and the formation of barrel walls.

### The ratio of barrel/septum size is decreased after *Cr* deletion

**Fig. 3:**
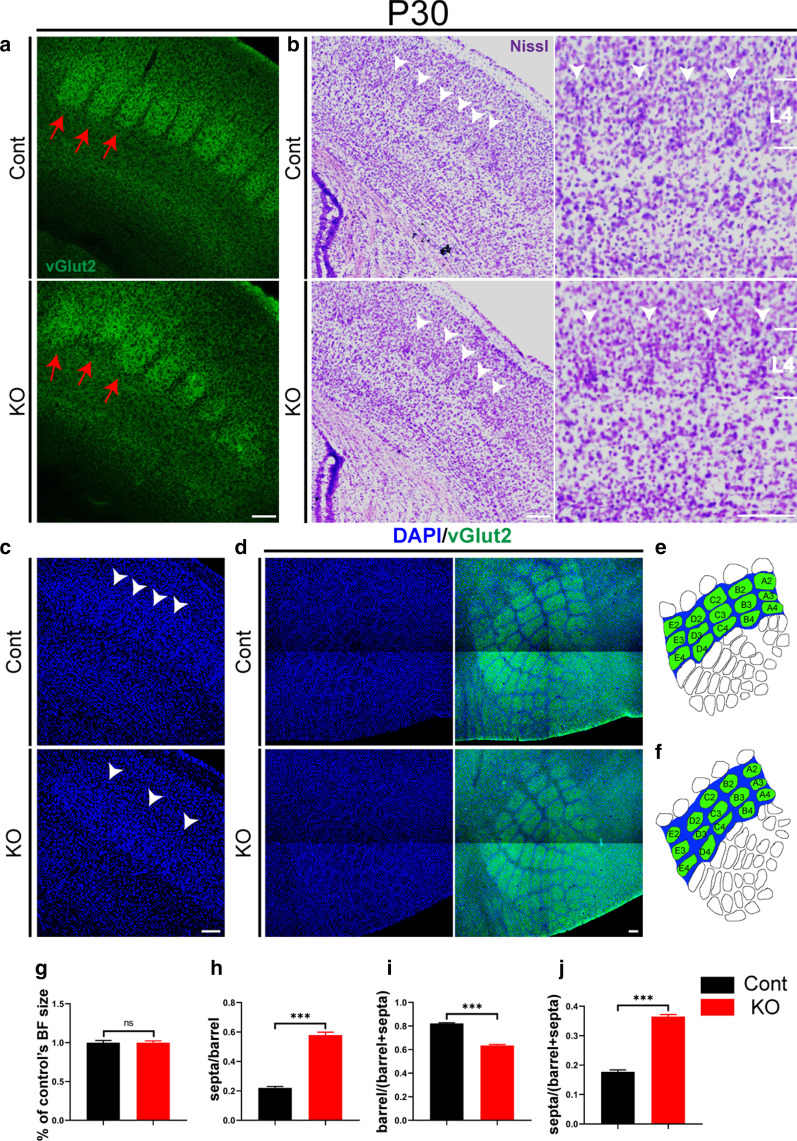
*Cr* deletion leads to a decreased ratio of barrel/septum size. **a** Immunostaining of vGlut2 in the P30 barrel cortex showed that in *Cr* KO mice, the organization of barrel columns was partly spread out compared with that in control mice (indicated by the red arrows; Cont, n = 3; KO, n = 3). **b** Nissl staining showed that the L4 spiny stellate neurons of *Cr* KO mice became disordered in the barrel wall regions compared with those of the control mice (indicated by the white arrowhead; Cont, n = 3; KO, n = 3). **c** DAPI staining further confirmed the disrupted arrangement of L4 spiny stellate neurons in barrel wall regions in *Cr* KO mice at P30 (indicated by the white arrowheads; Cont, n = 3; KO, n = 3). **d**–**f** Immunostaining of DAPI and vGlut2 in a flattened tangential section across L4 of the barrel cortex showing the entire pattern of the barrel field. The barrel field was reconstructed using ImageJ software. The morphometric data (from A2 to E4; the barrels are green, and the septa are blue) of the barrel field were analyzed (Cont, n = 3; KO, n = 3). **g**–**j** Morphometric analysis of the barrel field. Loss of *Cr* had no effect on the size of the entire barrel field. The ratio of septa/barrel size (from A2 to E4) was significantly increased in *Cr* KO mice compared with control mice. The decreased barrel/(barrel + septa) ratio and increased septa/(barrel + septa) ratio further confirmed that the barrel column was contractible and that the septa column was outstretched in *Cr* KO mice (Cont, n = 3; KO, n = 3). Data are presented as the mean ± SEM; unpaired Student’s t-test. ***p < 0.001. Scale bars: 200 μm.

To further investigate the effect of *Cr* deletion in L5a on the maturation of the barrel/septum microcircuit, we explored the morphology of the barrel field by immunostaining of vGlut2 at P30 when barrel cortex development was completed [43]. Control mice exhibited well-arranged barrels (Fig. 3a). *Cr* KO mice still displayed a disrupted arrangement pattern; furthermore, the individual barrel was more dispersed and indistinct (Fig. 3a). Nissl staining showed that at P30 in control mice, barrel walls clearly formed a “thin” wall. In *Cr* KO mice, barrel walls generally formed and were positioned in the expected area; however, it seemed that L4 spiny stellate neurons were distributed more broadly in *Cr* KO mice than in control mice (Fig. 3b), consistent with the widened intervals viewed by vGlut2 staining (Fig. 3a). This phenotype was further confirmed by DAPI staining (Fig. 3c).

We prepared flattened tangential cortical slices to examine the holonomic barrel field by double immunostaining of vGlut2 with DAPI [47, 51], and the entire barrel field was reconstructed and analyzed using ImageJ software (Fig. 3e, f). We calculated and compared the size of the entire barrel field and found that there was no remarkable difference in the size of the entire barrel field in *Cr* KO mice compared with control mice (Fig. 3g). We then explored the size of individual barrels and septa within the major mystacial whisker barrels (from A2 to E4). Our results showed that the barrel/septum ratio was significantly increased (Fig. 3h), indicating that the individual barrel columns were contractible and that the septa columns were correspondingly outstretched. This change was further confirmed by the decreased barrel/ (barrel + septum) ratio (Fig. 3i) and increased septum/ (barrel + septum) ratio (Fig. 3j). Taken together, these results indicate that CR is required for the normal formation of barrels and septum in the barrel cortex due to its regulation of the development of L5a pyramidal neuron dendrites.

### Both membrane excitability and excitatory synaptic transmission are increased in *Cr* KO L5a pyramidal neurons

**Fig. 4:**
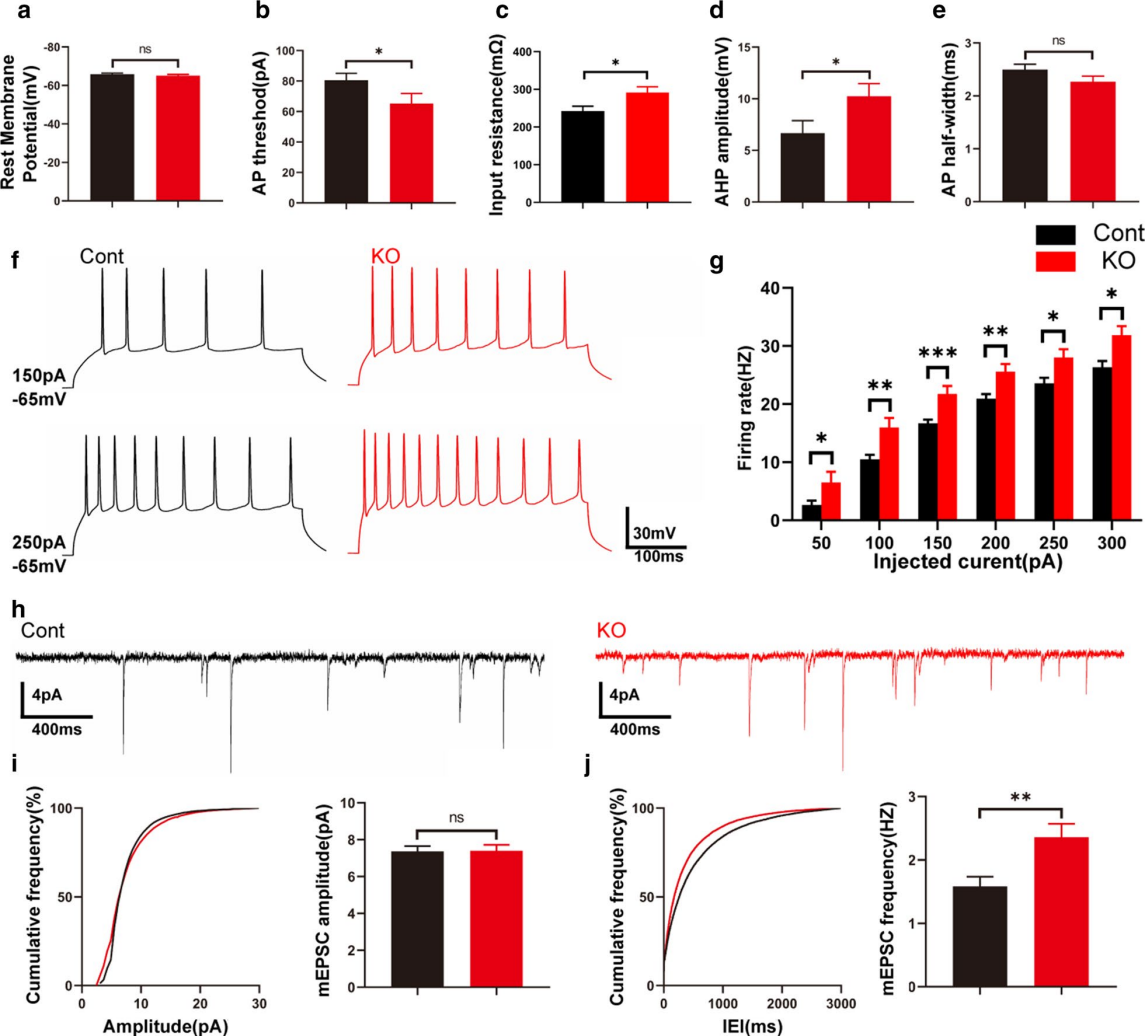
Increased neuronal excitability and excitatory synaptic transmission in L5a *Cr* KO neurons. **a**–**e** The resting membrane potentials in *Cr* KO L5a neurons were comparable to those in control L5a neurons (**a**). The mean action potential (AP) current threshold was markedly decreased in *Cr* KO neurons (**b**). Both the mean input resistance (**c**) and amplitude of the AHP (**d**) were significantly higher in *Cr* KO neurons than in control neurons. The changes in AP half-width (**e**) were not significantly different between control and *Cr* KO neurons (Cont, > 30 cells from 4 mice; KO, > 20 cells from 3 mice). **f** Representative responses to currents (150 and 250 pA) recorded from RFP^+^ L5a pyramidal neurons in the control (left, black) and *Cr* KO (right, red) mice. **g** The number of spikes is displayed against depolarizing current steps of increasing amplitude. Six 400-ms depolarizing currents ranging from 50 to 300 pA at 50-pA intervals were injected. *Cr* KO pyramidal neurons fired more action potentials than control neurons in response to the same amount of current (Cont, > 30 cells from 4 mice; KO, > 20 cells from 3 mice). **h** Sample traces of mEPSCs were recorded from RFP^+^ L5a pyramidal neurons of control and *Cr* KO mice. **i**–**j** The mean amplitude of mEPSCs (**i**) was not remarkably different between control and *Cr* KO neurons. However, the mean mEPSC frequency (**j**) was strongly increased in *Cr* KO neurons compared with control neurons (Cont, 25 cells from 4 mice; KO, 19 cells from 3 mice). Data are presented as the mean ± SEM; **a**–**e**, **g** unpaired Student’s t-test. **i**–**j**, K-S test. *p < 0.05; **p < 0.01; ***p < 0.001.

As an intrinsic Ca^2+^ buffer, CR plays an important role in the regulation of neuronal excitability and neurotransmitter release [25, 52]. We next performed whole-cell patch-clamp recording on RFP^+^ neurons using acute brain slices to investigate the effect of *Cr* deletion on the maturation and excitability of L5a pyramidal neurons. We investigated intrinsic cell electroresponsiveness through current-clamp recordings. The resting membrane potential was recorded immediately after perforating the cell membrane and was found to be comparable between control and *Cr* KO neurons (Fig. 4a) [37]. However, a significant decrease in the action potential current threshold was detected after *Cr* deletion (Fig. 4b). Moreover, the mean input resistance and amplitude of the AHP were significantly higher in *Cr* KO neurons than in control neurons (Fig. 4c, d). Although the action potential half-width showed a slight tendency to decrease, there was no statistical significance between control and *Cr* KO neurons (Fig. 4e). Unsurprisingly, in response to a series of suprathreshold depolarizing current injections with amplitudes ranging from − 50 to 300 pA (with an increment of 50 pA), the number of action potentials recorded from *Cr* KO neurons was significantly higher than that of the control neurons (Fig. 4f, g).

To assess the functional consequences of *Cr* deletion on synaptic transmission, we next tested the basic synaptic transmission of L5a pyramidal neurons and compared them with the amplitude and frequency of spontaneous miniature EPSCs (mEPSCs) (Fig. 4h) [53]. BMI and TTX were applied to block GABA receptor-mediated inhibitory currents and action potential-dependent synaptic transmission, respectively. We found that the mean amplitude of mEPSCs was unaffected (Fig. 4i), while the mean frequency was strongly increased in *Cr* KO neurons compared with control neurons (Fig. 4j). Collectively, these results suggest that loss of *Cr* leads to increased membrane excitability and excitatory synaptic transmission of L5a pyramidal neurons.

### *Cr* KO mice exhibit pronounced exploratory behavior deficits

**Fig. 5:**
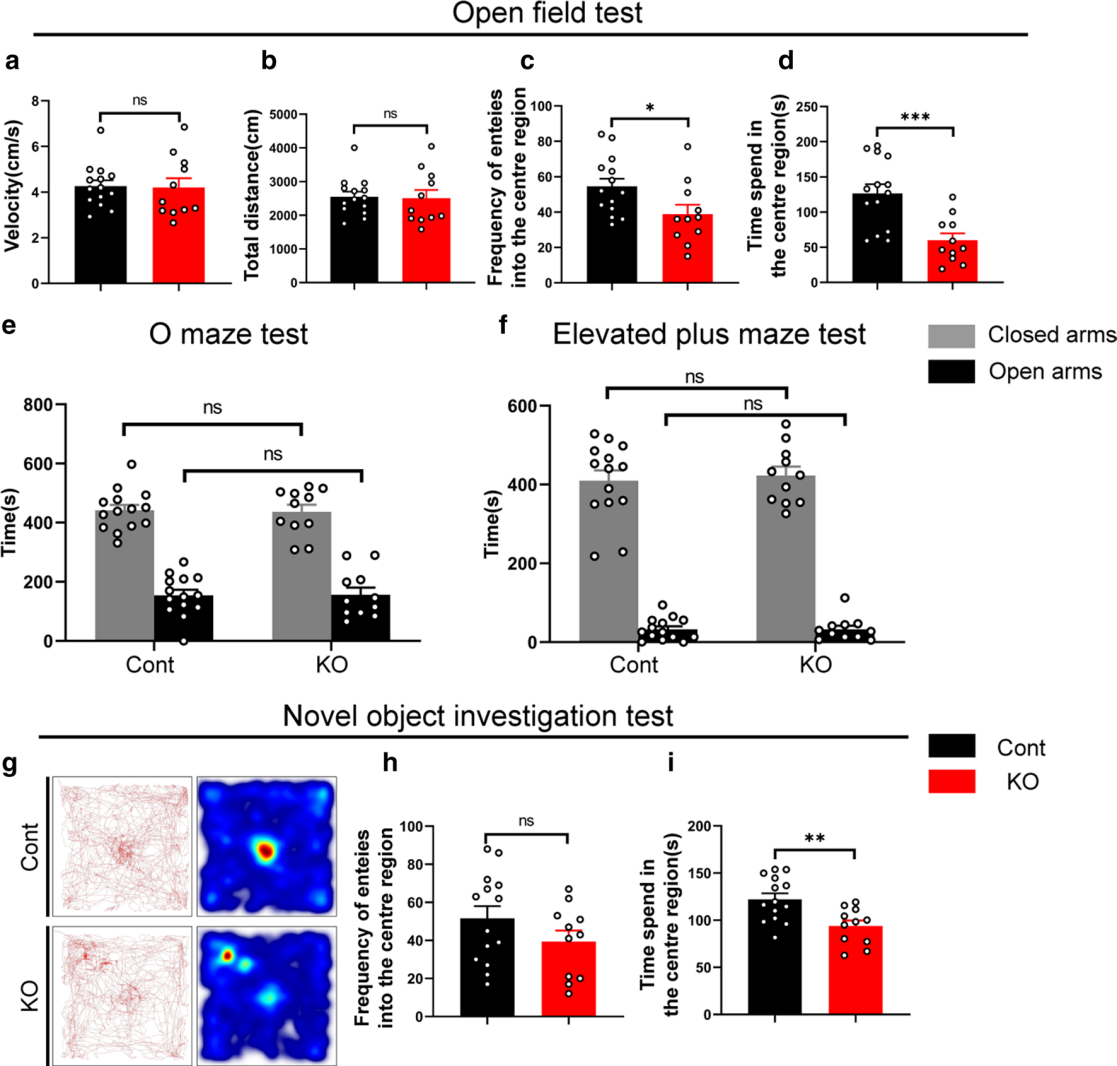
*Cr* KO mice exhibit pronounced exploratory behavior deficits. **a**–**b** The average velocity (**a**) and total distance (**b**) traveled in 30 min were similar between control and *Cr* KO mice during the open-field test. **c**–**d** The frequency of entries (**c**) and duration (**d**) in the center zone during the first 5 min in the open-field test were significantly decreased in *Cr* KO mice. **e**–**f** The comparable open-arm times observed in the elevated O-maze (**e**) and elevated plus maze (**f**) tests indicated that *Cr* KO mice had normal anxiety levels. **g** Representative movement paths of control and *Cr* KO mice during the novel object investigation test. **h**–**i** The frequency of entering the center zone (**h**) was comparable between control and *Cr* KO mice. *Cr* KO mice spent significantly less time investigating the object than control mice (**i**). Data are presented as the mean ± SEM; unpaired Student’s t-test. Cont, n = 14; KO, n = 11. *p < 0.05; **p < 0.01; ***p < 0.001

Rodents use their whiskers as multipurpose organs for behaviors ranging from object detection, including object localization, judgment of shape and texture, and discrimination, to movement coordination, such as detecting distance and motor coordination [1–3, 6]. Since L5a plays a crucial role in integrating information resources and coordinating the movement of the whiskers [9, 30], we performed a series of behavioral tests related to the barrel cortex. Considering the rodents use their whiskers to explore the surroundings when moving through a new environment, we first conducted an open-field test to evaluate the spontaneous spatial exploration ability [1]. We found that the mean velocity and the total distance traveled within a 30-min duration were comparable between *Cr* KO and control mice (Fig. 5a, b), indicating that locomotor activity was unaffected. However, within the first five minutes, the time spent in the center zone and the frequency of entering the center zone were obviously decreased in *Cr* KO mice (Fig. 5c, d). This result suggested two possibilities: increased anxiety or decreased desire to explore.

To further examine the level of anxiety, we conducted elevated O-maze and elevated plus maze tests [34, 54]. As shown in Fig. 5e and f, there were no significant differences in the time spent in the open arms between the control and *Cr* KO mice, demonstrating that loss of *Cr* had no influence on anxiety levels. To test the exploratory behaviors, a novel object was introduced to the center of the open field after the mice became fully familiar with the environment [40]. As shown in Fig. 5g, the trajectories of control mice were more concentrated in the central area around the novel object than those of *Cr* KO mice. Although the frequency of entering the center zone was comparable (Fig. 5h), *Cr* KO mice spent significantly less time investigating the object than control mice (Fig. 5i). Taken together, these data indicate that deletion of *Cr* in L5a has no effects on spontaneous motor ability but impairs exploratory behaviors.

### *Cr* KO mice display defects in whisker-associated tactile sensation behavior

**Fig. 6:**
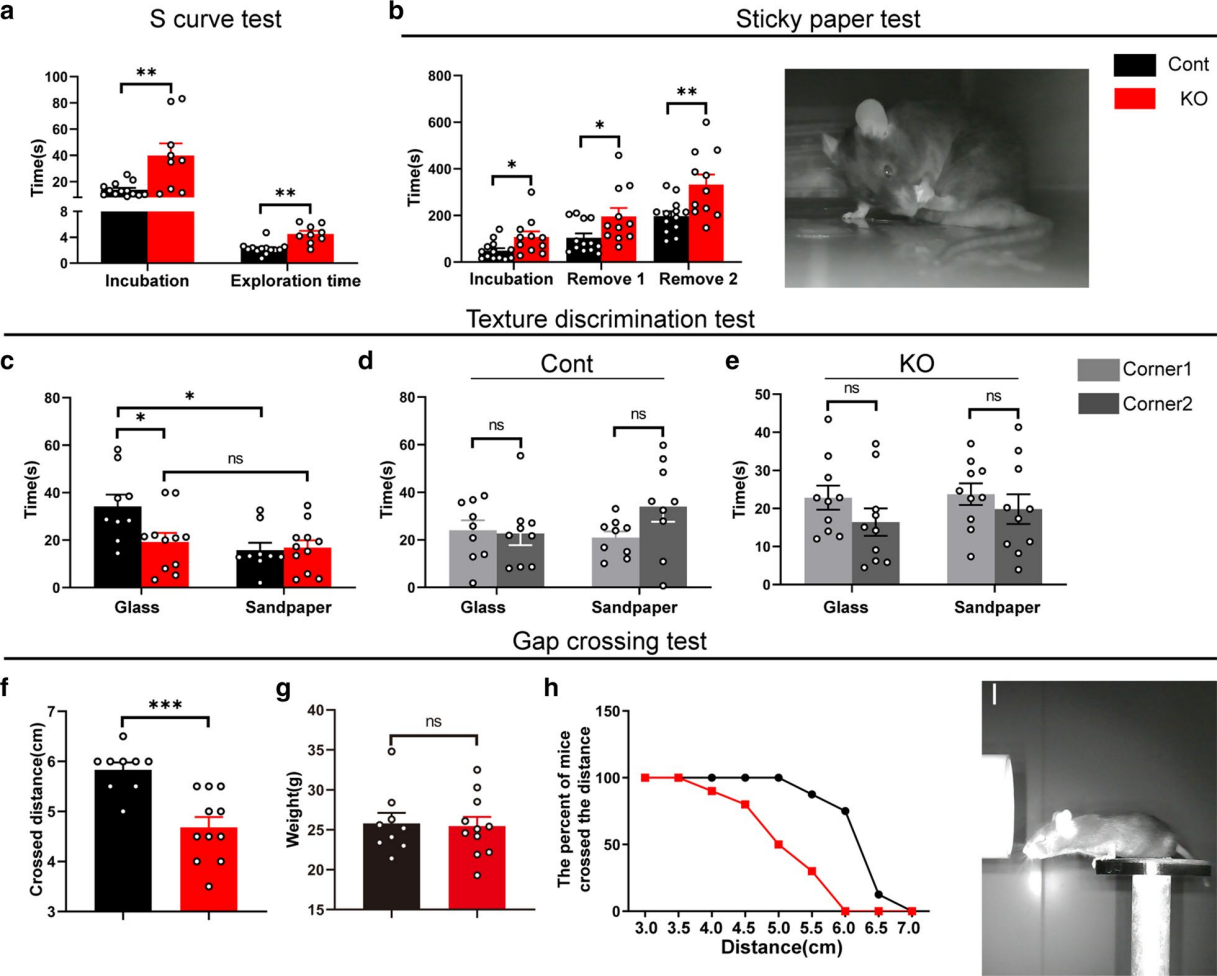
*Cr* removal impairs whisker-associated tactile sensation behavior. **a** *Cr* KO mice needed more time to reach the destination (incubation) and spent more time exploring the opening area before exiting the curve in the S curve test (exploration time; Cont, n = 13; KO, n = 8). **b** *Cr* KO mice spent more time finding the adhesive paper stuck on the palmar surface of their hind paws (incubation) and spent more time removing the paper (Cont, n = 13; KO, n = 11). **c**–**e** In the texture discrimination test, the control mice preferred to stay with the smooth glass bottle, but the *Cr* KO mice did not exhibit any preference for either corner (**c**). Neither control nor *Cr* KO mice exhibited any preference for either corner when identical bottles were presented (**d**, **e**; Cont, n = 9; KO, n = 11). **f**–**i** The distances crossed by *Cr* KO mice in the gap-crossing test were significantly shorter than those crossed by control mice (**f**); even though the groups had identical weights (**g**), the control mice performed better than the *Cr* KO mice (**h**). This test was performed under infrared lighting conditions (**i**; Cont, n = 9; KO, n = 11). Data are presented as the mean ± SEM. **a**–**b** and **d**–**g**, unpaired Student’s t-test. **c** Multiple Student’s t-test with Bonferroni correction. *p < 0.05; **p < 0.01; ***p < 0.001

To further investigate exploratory behavior deficits, we performed an “S” curve test. This test is designed to simulate a narrow nocturnal environment. When the mice were introduced into an unfamiliar curve, they explored the curve with their whiskers while moving forward [6]. *Cr* KO mice spent more time going through the “S” curve to reach their destination (incubation) than the control mice (Fig. 6a); moreover, *Cr* KO mice also spent more time exploring the opening area near the exit before leaving the curve (Fig. 6a).

To measure tactile responses after *Cr* deletion in L5a, we performed a sticky paper test [39, 41]. This test is designed to measure tactile responses to adhesive paper stuck on the palmar surface of mouse hind paws. Mice were required to detect the tape with the help of whiskers and then to remove it. The results showed that *Cr* KO mice spent more time finding the tape (incubation); accordingly, they spent more time removing the tape from their hind paws (Fig. 6b).

Next, we examined accurate recognition ability through a texture discrimination test [1, 55, 56]. Mice were first allowed to move freely to explore different textures in the open field box. Ten minutes later, two glass bottles, one of which was wrapped in sandpaper, were placed in the opposite corners of the box. The time that mice stayed in each corner in the next ten minutes was assessed. We found that the control mice spent more time in the corner where the unwrapped glass bottle was placed, while the *Cr* KO mice did not exhibit any preference for the two corners (Fig. 6c), suggesting that the texture discrimination of *Cr* KO mice was impaired. We also assessed the time that *Cr* KO mice stayed with two identical bottles and found that they had no position preference (Fig. 6d, e).

Finally, we performed the gap crossing test [39, 42], a specific test to detect the distance perception ability of cortical whiskers. It consists of a series of trials requiring the mice to accurately measure gaps of variable distances and to cross the gaps to reach a safe platform (Fig. 6i). The average gap distance crossed in *Cr* KO mice was significantly shorter than that in control mice (Fig. 6f). Weight data confirmed that the disparity was not related to body size (Fig. 6g). As the gap distances increased, the percentage of mice that were able to cross the gap gradually decreased (Fig. 6h). Collectively, our data suggest that tactile sensation was impaired in *Cr* KO mice.

### Deletion of *Cr* impairs social novelty preference

**Fig. 7:**
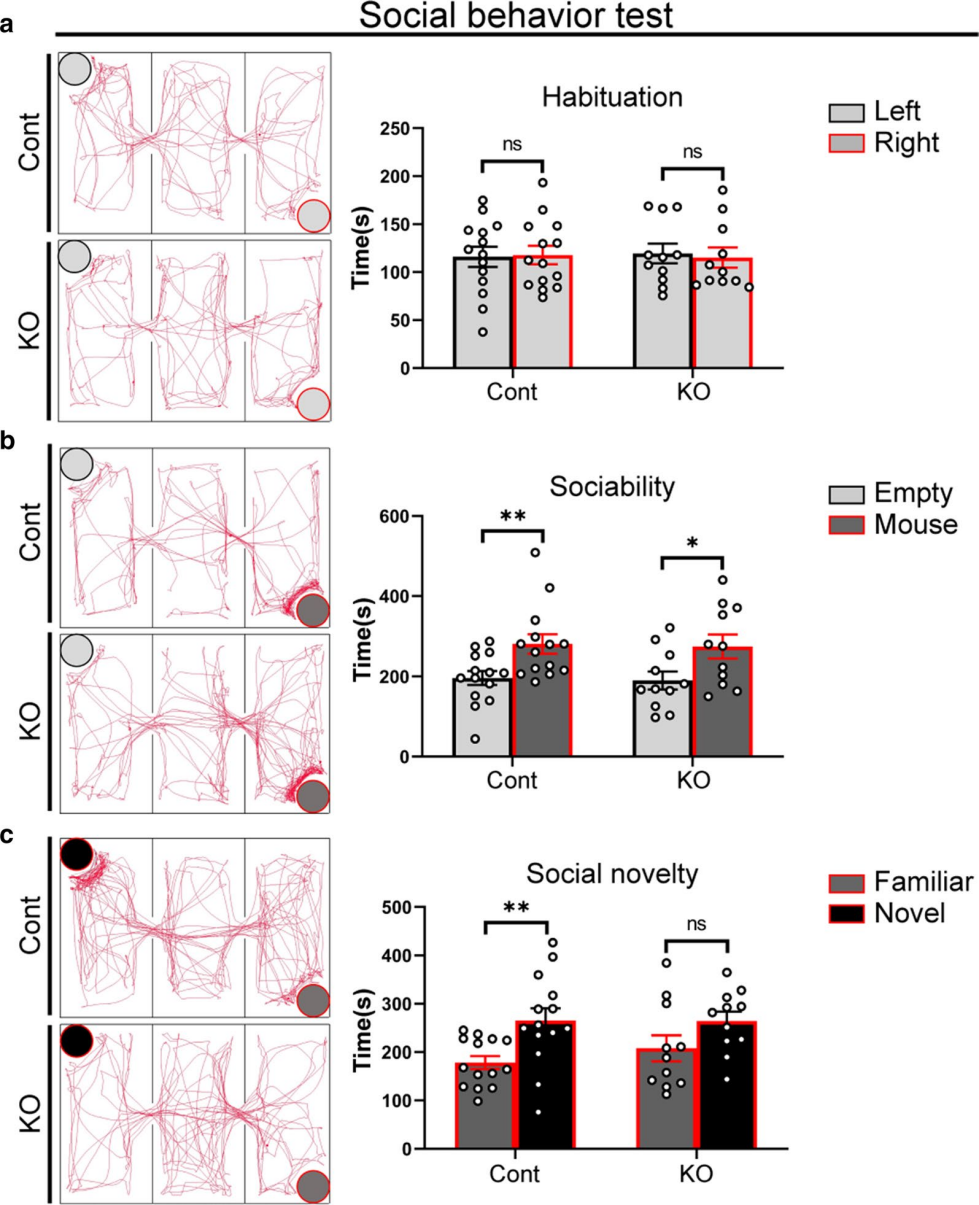
Disruption of *Cr* results in impaired social novelty preference. **a** Neither control nor *Cr* KO mice showed a preference for either of the two lateral chambers in the habituation phase of the social behavior test. **b** All mice spent more time with the first stimulating mouse during the social recognition phase. **c** *Cr* KO mice did not show any preference during the social novelty phase. Data are presented as the mean ± SEM. **a**–**c**, unpaired Student’s t-test. Cont, n = 14; KO, n = 12. *p < 0.05; **p < 0.01

In addition to discriminating simple tactile properties, in social interaction, mice are able to get acquainted with each other and distinguish the social hierarchies though their facial whiskers [3, 57]. To investigate social behavior deficits, we performed the three-chamber test, and found that neither control nor *Cr* KO mice displayed a preference for either of the two empty chambers during the habituation phase (Fig. 7a), and they all spent more time with the first stimulating mouse (Fig. 7b), indicating that the loss of *Cr* had no effect on social ability. In the social novelty test, control mice displayed a preference for the novel mouse, while *Cr* KO mice did not show any preference (Fig. 7e), suggesting that loss of *Cr* impairs social novelty preference. In summary, loss of *Cr* affects the normal formation of the barrel and septa columns in the barrel cortex so that tactile information cannot be processed properly, leading to related behavioral deficits.

## Discussion

In this study, we show that deletion of *Cr* in L5a pyramidal neurons resulted in a decrease in dendritic complexity. Importantly, L5a dendritic deficits subsequently led to abnormal formation of the barrels and barrel walls. Moreover, the membrane excitability and excitatory synaptic transmission of L5a neurons were increased. C*r* KO mice exhibited pronounced exploratory and whisker-associated tactile sensation behavioral deficits. Our results demonstrate that CR expressed in L5a is important for the development of the barrel cortex, including both the barrel and septa columns.

### L5a and the formation of the barrel and the barrel wall

In the barrel cortex, L5a pyramidal neurons are involved in both the barrel and septa circuit and considered to be an important integration site of the lemniscal and paralemniscal pathways [15, 17]. The development of the barrel cortex is an activity- and experience-dependent process [22, 44, 45]. L5a pyramidal neurons receive a strong direct input from L4 barrels and preferentially establish synaptic connections with cells in the L4 barrel wall region [14, 16, 18]. Previously, we reported that CR is dynamically expressed in L5a pyramidal neurons during the key developmental window of the barrel cortex [30]. In this study, we found that the length and complexity of L5a pyramidal neuron dendrites were significantly decreased in *Cr* KO mice. Moreover, in contrast to the dense barrel wall observed in the control mice, L4 spiny stellate cells could not properly gather, leading to undetectable barrel walls at P8 and thickened barrel walls at P30 in *Cr* KO mice. To our knowledge, this study is the first to morphologically show changes in the accumulation of L4 spiny stellate cells, which may be a direct consequence of failed/fewer connections between L5a and L4 neurons. It will be interesting to explore how the information flow contributes to the formation of the barrel wall.

### CR in the excitability of L5a pyramidal neurons

Ca^2+^ is required for numerous cellular functions [25]. As a Ca^2+^-binding protein, CR regulates intracytoplasmic Ca^2+^ concentration though direct “binding”; moreover, as a Ca^2+^ sensor, CR controls the distribution of Ca^2+^ though its spontaneous activity, thus affecting a series of physiological activities [26–28]. Here, we show that deletion of *Cr* led to an increase in both the neuronal excitability and the excitatory synaptic transmission of L5a pyramidal neurons. Thus, our results are congruent with previous reports of increased neuronal excitability in cerebellar granule cells lacking CR.

### Abnormal exploratory behavior and whisker-associated tactile sensation behavior in *Cr* KO mice

As nocturnal animals, mice navigate in the dark and explore objects largely with their whiskers [2, 3]. The whisker-barrel cortex system executes tasks such as estimating spatial orientation, object positioning and texture discrimination as well as gap distance measurement [4, 42]. The afferent information from the lemniscal and paralemniscal pathways converges on L5a pyramidal neurons [9]. L5a pyramidal neurons also receive encoded information from other laminae and convey information to the secondary somatosensory (SII) cortex, primary motor (MI) cortex, contralateral barrel cortex and subcortical motor regions [6, 9]. In this study, *Cr* KO mice exhibited exploratory behavior deficits and were unable to estimate gap distance accurately. They also show impaired tactile sensation. These findings expand our knowledge of L5a pyramidal neurons in the whisker-barrel cortex system. In addition to L5a pyramidal neurons, CR is also expressed in several other neuron types which may also contribute to behavioral impairments which observed in this study, it will be interesting to explore the specific roles of CR in distinct neuron types by using conditional disruption strategy in the future.

## Supplementary Information

**Additional file 1:** Raw data supplementary materials.

**Additional file 2:** Statistical results of raw data.

## Data Availability

The data generated or analyzed during this study are included in this published article.
