# Supplementary material for: Loss of Calretinin in L5a impairs the formation of the barrel cortex leading to abnormal whisker-mediated behaviors
Source: Mol Brain. 2021 Apr 12;14:67. doi: 10.1186/s13041-021-00775-w (PMC8042711; doi:10.1186/s13041-021-00775-w)
Supplement: Supplementary file 1 — Additional file 1: Raw data supplementary materials. [file 13041_2021_775_MOESM1_ESM.pdf]

Raw Data Supplementary Materials

Figure 1D-F Raw Data. Immunostaining against RFP showed that CR^{+} L5a pyramidal neurons were labeled by RFP. The lengths of apical dendrites, basal dendrites and total dendrites were significantly reduced in *Cr* KO neurons, and the complexity of dendrites in *Cr* KO mice was markedly decreased. Quantified data was listed in Raw Data Excel sheet1. Cont, 21 cells from 3 mice; KO, 27 cells from 4 mice. Scale bar, 100 μm.

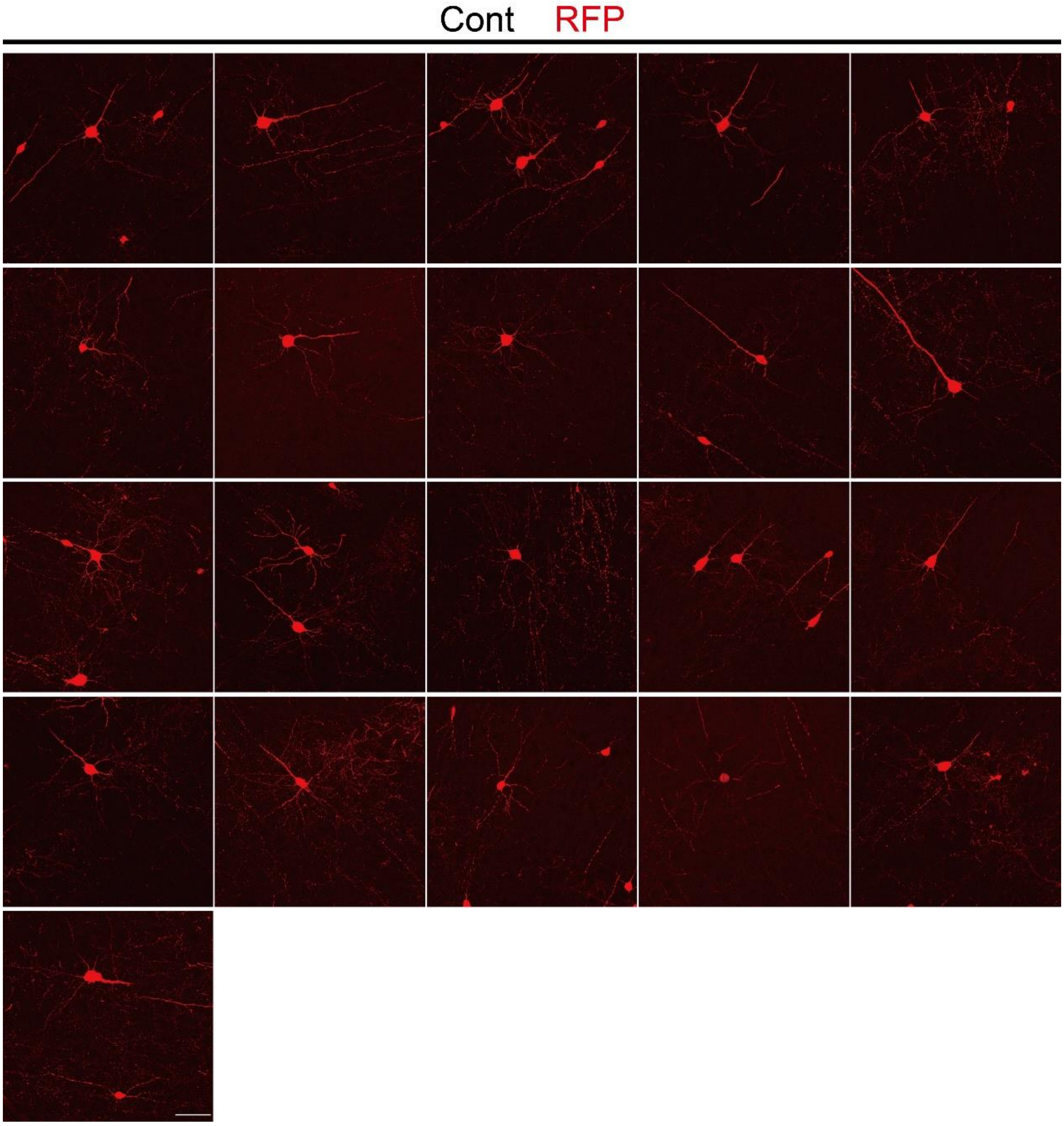

KO RFP

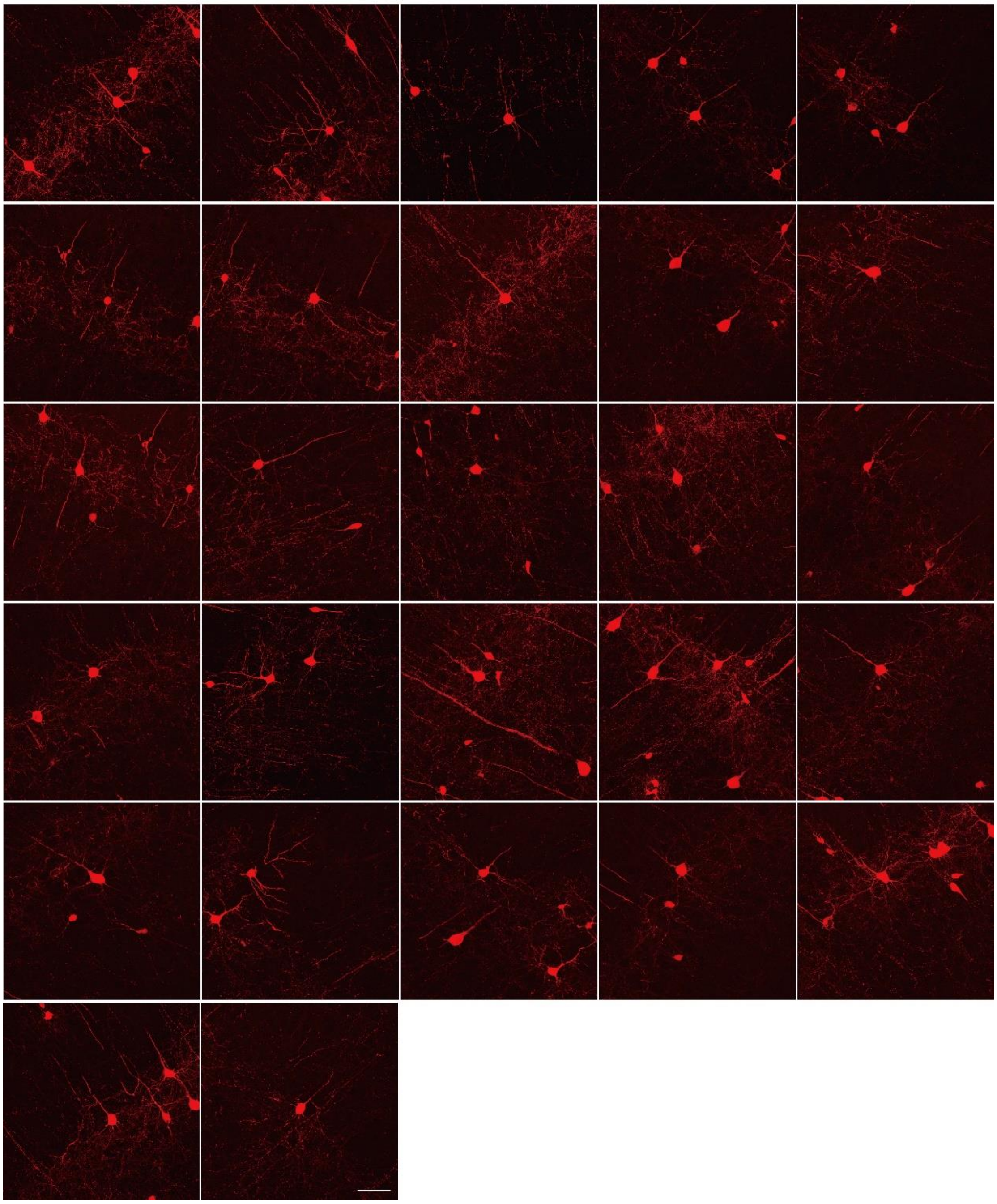

Figure 2E Raw Data. Immunostaining of vGlut2 in the P8 barrel cortex showed that the organization of the barrels was disordered in *Cr* KO mice (indicated by the red arrows). Cont, n = 4; KO, n = 4. Scale bar, 200 μm.

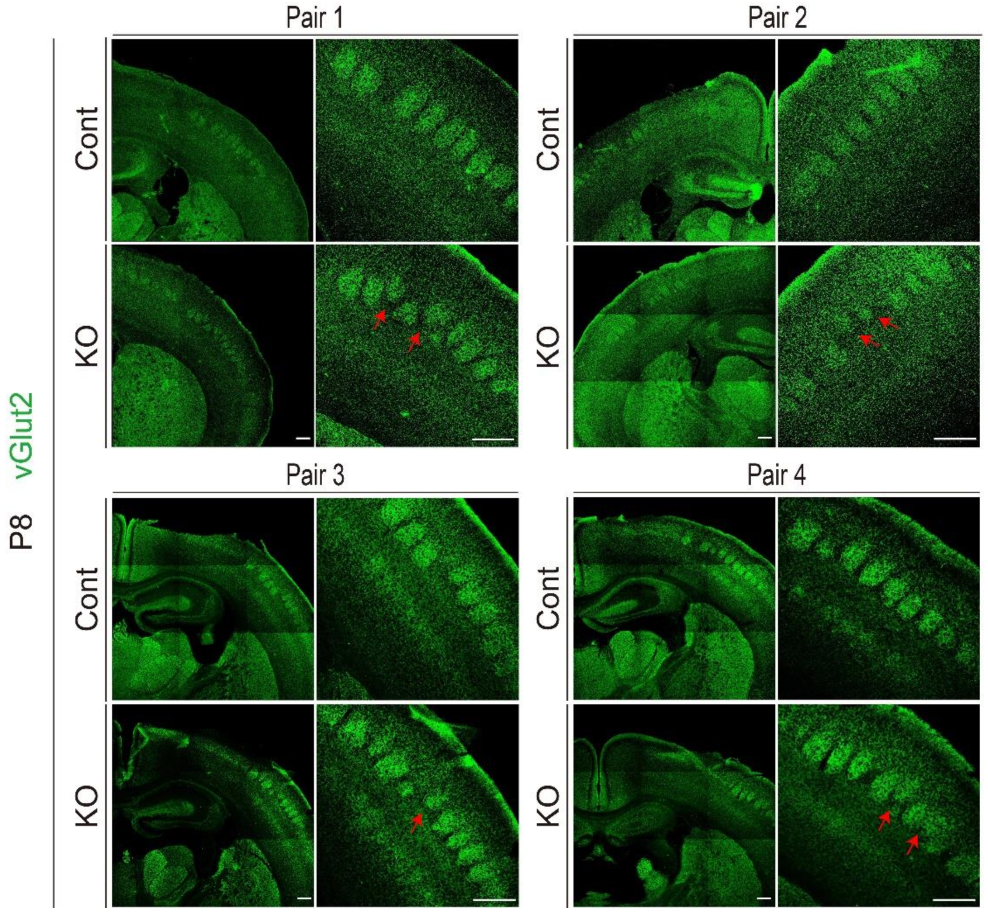

Figure 2F Raw Data. Nissl staining at P8 showing that, in contrast to the clear thin barrel wall observed in the control barrel cortex, abnormally gathered spiny stellate cells were observed in L4 of the *Cr* KO cortex (indicated by the white arrowheads). Cont, n = 4; KO, n = 4. Scale bar, 200 μm.

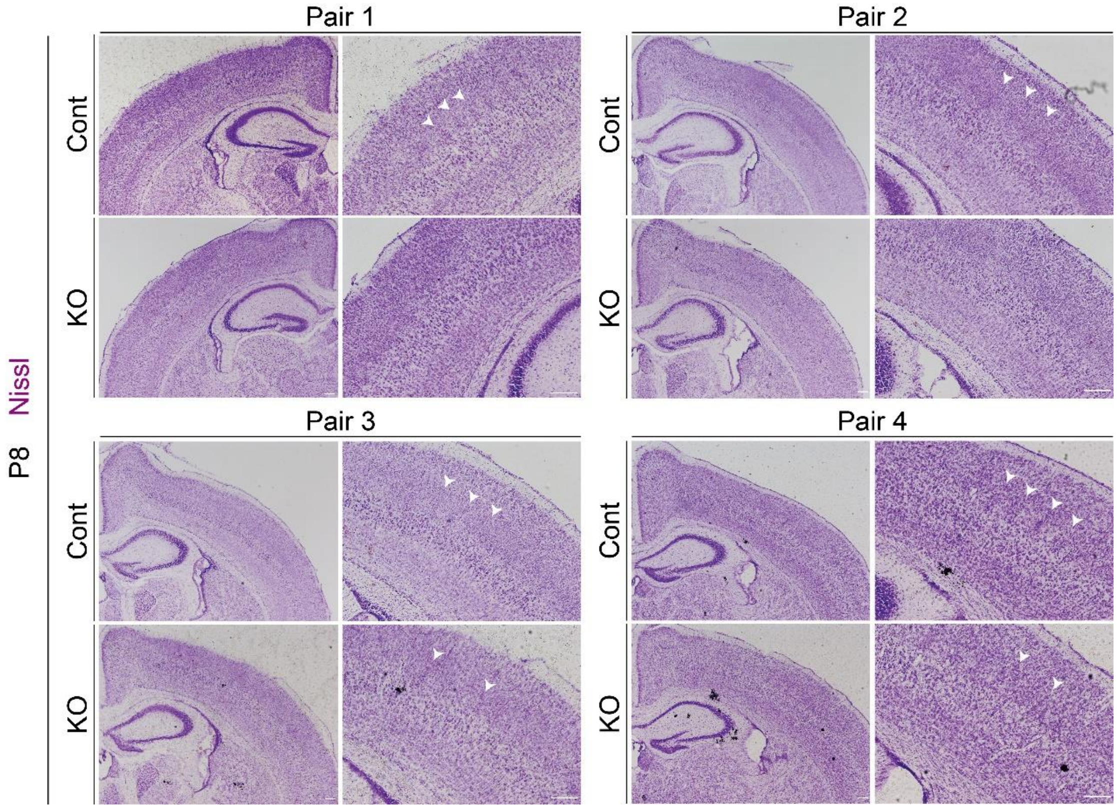

Figure 3A, C Raw Data. Immunostaining of vGlut2 in the P30 barrel cortex showed that in *Cr* KO mice, the organization of barrel columns was partly spread out compared with that in control mice (indicated by the red arrows). DAPI staining confirmed the disrupted arrangement of L4 spiny stellate neurons in barrel wall regions in *Cr* KO mice at P30 (indicated by the white arrowheads). Cont, n = 3; KO, n = 3. Scale bar, 200 μm.

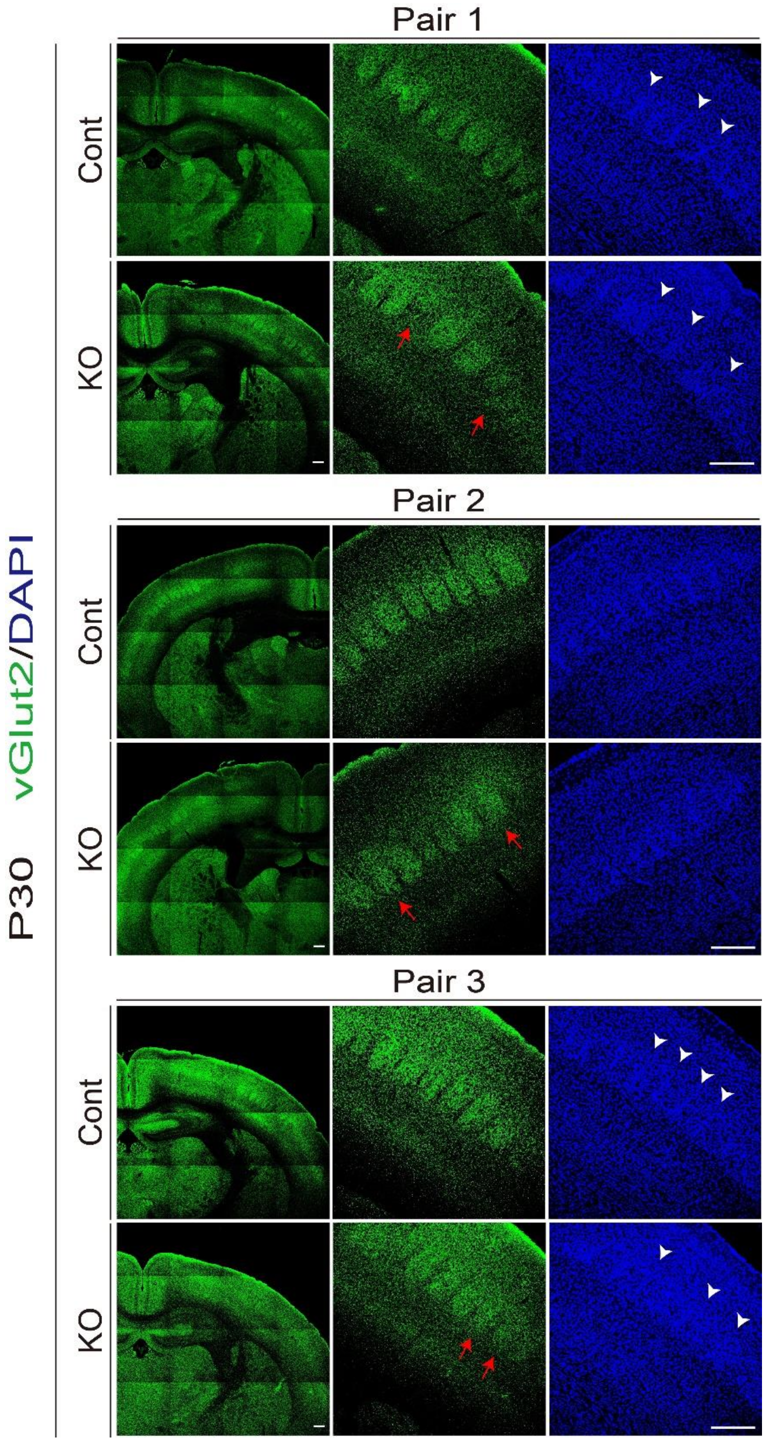

Figure 3B Raw Data. Nissl staining showed that the L4 spiny stellate neurons of *Cr* KO mice became disordered in the barrel wall regions compared with those of the control mice (indicated by the white arrowhead). Cont, n = 3; KO, n = 3. Scale bar, 200 μm.

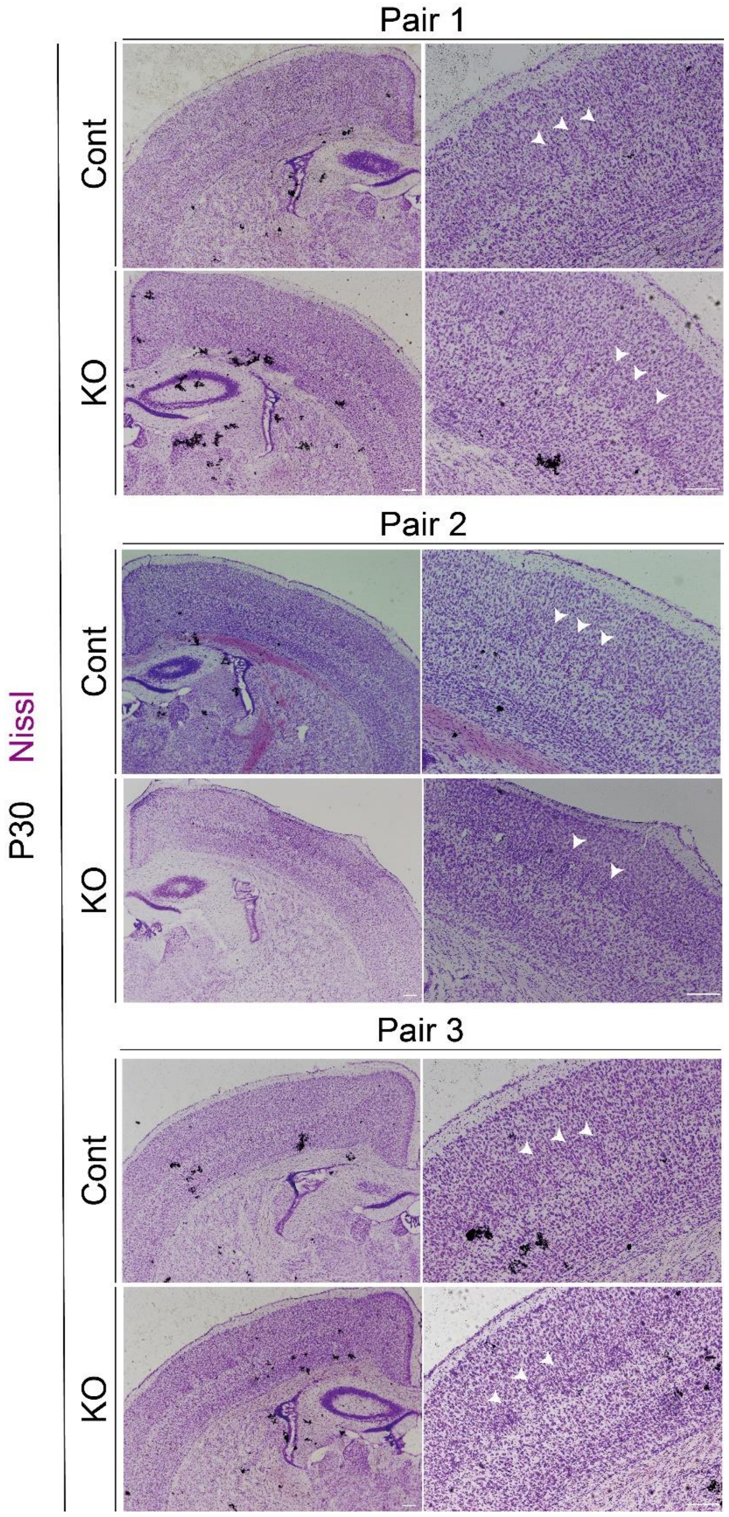

Figure 3D-J Raw Data. Immunostaining of DAPI and vGlut2 in a flattened tangential section across L4 of the barrel cortex showing the entire pattern of the barrel field. Quantified data of morphometric analysis of the barrel field was listed in Raw Data Excel sheet2. Cont, n = 3 (pair1-3).; KO, n = 3 (pair1-3). Scale bar, 200 μm.

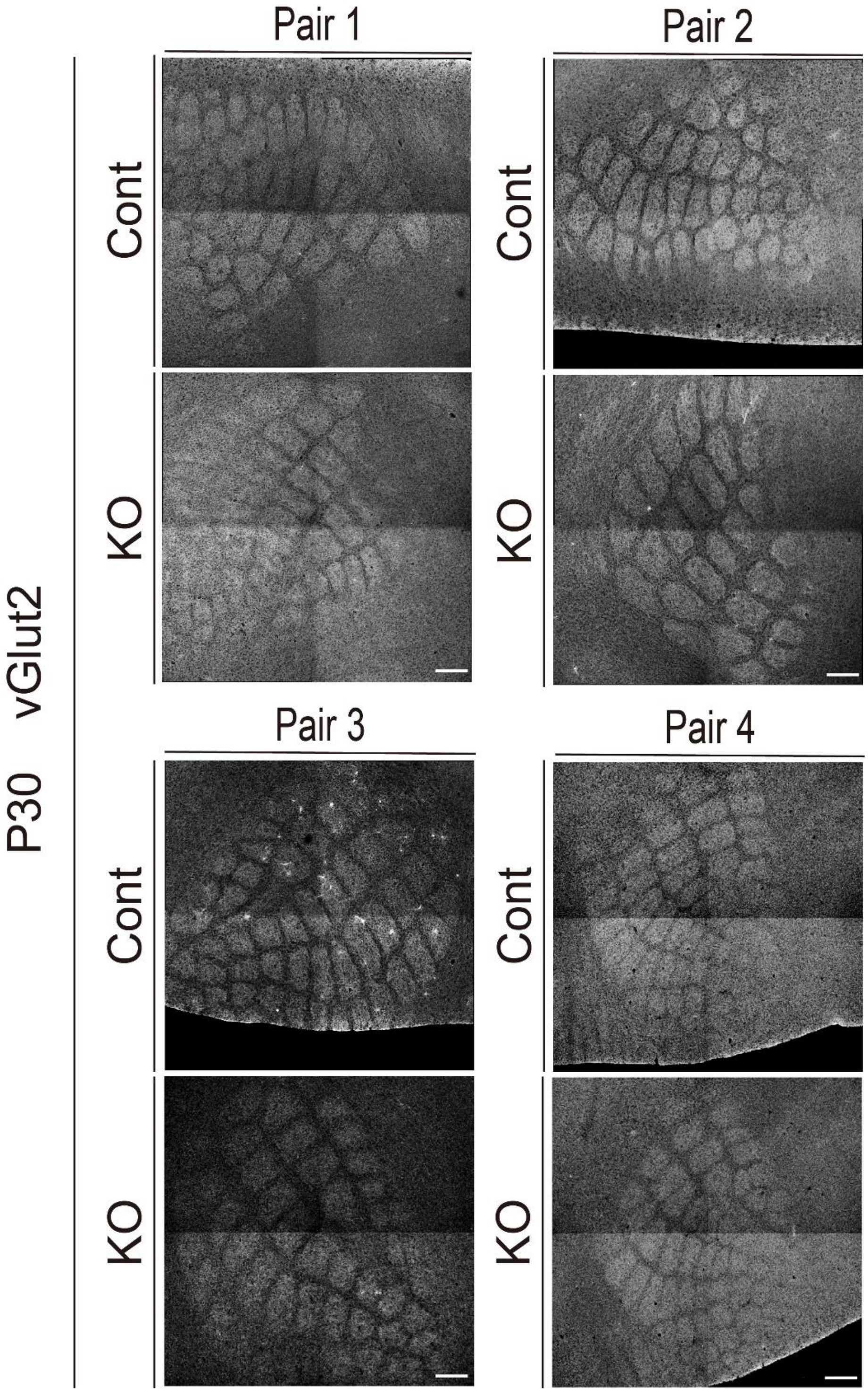

Figure 4A-G Raw Data. Increased neuronal excitability in L5a *Cr* KO neurons. Quantified data was listed in Raw Data Excel sheet3. Cont, > 30 cells from 4 mice; KO, > 20 cells from 3 mice.

Figure 4H-J Raw Data. Increased excitatory synaptic transmission in L5a *Cr* KO neurons. Quantified data was listed in Raw Data Excel sheet4. Cont, 25 cells from 4 mice; KO, 19 cells from 3 mice.

Figure 5A-D Raw Data. The average velocity and total distance traveled in 30 mins were similar between control and *Cr* KO mice during the open-field test. The frequency of entries and duration in the center zone during the first 5 mins in the open-field test were significantly decreased in *Cr* KO mice. Quantified data was listed in Raw Data Excel sheet5. Cont, n = 14; KO, n = 11.

Figure 5E-F Raw Data. The comparable open-arm times observed in the elevated O-maze and elevated plus maze tests indicated that *Cr* KO mice had normal anxiety levels. Quantified data was listed in Raw Data Excel sheet6 and sheet6. Cont, n = 14; KO, n = 11.

Figure 5H-I Raw Data. The frequency of entering the center zone was comparable between control and *Cr* KO mice. *Cr* KO mice spent significantly less time investigating the object than control mice. Quantified data was listed in Raw Data Excel

sheet8. Cont, n = 14; KO, n = 11.

Figure 6A Raw Data. *Cr* KO mice needed more time to reach the destination (incubation) and spent more time exploring the opening area before exiting the curve in the S curve test. Quantified data was listed in Raw Data Excel sheet9. Cont, n = 13; KO, n = 8.

Figure 6B Raw Data. *Cr* KO mice spent more time finding the adhesive paper stuck on the palmar surface of their hind paws (incubation) and spent more time removing the paper. Quantified data was listed in Raw Data Excel sheet10. Cont, n = 13; KO, n = 11.

Figure 6C-E Raw Data. In the texture discrimination test, the control mice preferred to stay with the smooth glass bottle, but the *Cr* KO mice did not exhibit any preference for either corner. Neither control nor *Cr* KO mice exhibited any preference for either corner when identical bottles were presented. Quantified data was listed in Raw Data Excel sheet11. Cont, n = 9; KO, n = 11.

Figure 6F-H Raw Data. The distances crossed by *Cr* KO mice in the gap-crossing test were significantly shorter than those crossed by control mice; even though the groups had identical weights, the control mice performed better than the *Cr* KO mice. Quantified data was listed in Raw Data Excel sheet12. Cont, n = 9; KO, n = 11.

Figure 7A-C Raw Data. Disruption of Cr results in impaired social novelty preference. Quantified data was listed in Raw Data Excel sheet13. Cont, n = 14; KO, n = 12.
